# Exploring the Mechanism of Ionic Liquids to Improve the Extraction Efficiency of Essential Oils Based on Density Functional Theory and Molecular Dynamics Simulation

**DOI:** 10.3390/molecules27175515

**Published:** 2022-08-27

**Authors:** Xiaorong Luo, Fen Wang, Guihua Wang, Hui Li

**Affiliations:** 1 China Resources Jiangzhong Pharmaceutical Group Co., Ltd., Nanchang 330006, China; 2 School of Pharmacy, Jiangxi University of Traditional Chinese Medicine, Nanchang 330004, China; 3 Institute of Chinese Materia Medica, China Academy of Chinese Medical Sciences, Beijing 100700, China; 4 Institute of Traditional Chinese Medicine Health Industry, China Academy of Chinese Medical Sciences, Nanchang 330000, China; 5 Jiangxi Health Industry Institute of Traditional Chinese Medicine, Nanchang 330000, China

**Keywords:** essential oil, ionic liquids, multivariate analysis, density functional theory, molecular dynamics simulations

## Abstract

**Highlights:**

- According to the design of the experiment (DoE), multivariate analysis models were used to optimize the critical process parameters combined with multi-objective optimization.
- Based on the optimized operating conditions, the MILT-HD method not only enhances the extraction efficiency from *Amomi fructus* but also reduces energy demands and CO_2_ emissions.
- Based on the density functional theoretical (DFT) and molecular dynamics (MD) simulations, the mechanisms for ionic liquids (ILs) to improve the extraction efficiency of essential oil was comprehensively revealed.

**Abstract:**

In this paper, *Amomi fructus* (Latin) was used to explore the mechanism of ionic liquids (ILs) in improving the extraction efficiency of essential oils. Microwave assisted ionic liquid treatment followed by a hydro-distillation (MILT-HD) process for isolating *Amomi fructus* essential oil was optimized by multi-objective optimization. Under optimum operating conditions, the IL-assisted extraction method not only enhances extraction efficiency but also reduces energy demands and CO_2_ emissions. Since the hydrogen bond structure network of cellulose in the cell wall is an important reason for hindering diffusion of essential oils, the mechanism of ILs was explored by density functional theoretical (DFT) and molecular dynamics (MD) simulations. According to DFT calculations, ILs can facilitate the cleavage of cellulose chains and have strong non-covalent interactions with cellulose. Based on the MD simulations, the degree of destruction of the cellulose hydrogen bond structure was explored. According to the DFT and MD simulations, the ILs can significantly destroy cellulose structure, thereby promoting essential oil release from the plant. These results were confirmed by scanning electron microscopy (SEM) and Fourier transform infrared spectroscopy (FTIR). This work is conducive to better understand the MILT-HD process for isolating essential oil and comprehensively understand the mechanism of ILs in the extraction process.

## 1. Introduction

*Amomi fructus*, as one of the top four authentic Traditional Chinese Plant Medicines [1], has a centuries-old history in medicine. *Amomi fructus* was also approved as a food by the China Food and Drug Administration (CFDA) [2]. According to Traditional Chinese Medicine (TCM) theory, there were 405 different drug products of *Amomi fructus* in prescription form registered with the CFDA [3]. Essential oils, as the principal active chemical component in *Amomi fructus*, has many biological activities such as anti-fungal and anti-bacterial properties [4], protective effects on gastric mucosa [5], anti-inflammatory and analgesic effects [4,6,7]. However, the traditional extraction methods, such as hydro-distillation (HD), for extracting essential oils have difficulty in meeting the increasing market demands. Moreover, this extraction method is inefficient, time-consuming and energy-consuming.

Ionic liquids (ILs), as cost-effective and environmentally benign solvents, were widely used to extract essential oils from plants with very high efficiency. Since ILs have an excellent capacity for effectively absorbing microwave energy, they can significantly enhance extraction efficiency [8]. Consequently, microwave-assisted ionic liquids treatment followed by hydro-distillation (MILT-HD) has shown many advantages. For example, Jiao et al. used the MILT-HD method to quickly and efficiently isolate the essential oil of *Dryopteris fragrans* [9]. However, there is still a lack of study on the mechanism of ILs in improving the extraction efficiency of essential oils from plants. In order to deeper understand the role of ILs in the extraction process, it is necessary to explore the mechanism of ILs.

For understanding ILs’ mechanism, some authors speculated the possible reasons for ILs to increase the extraction efficiency of essential oils [9,10,11]. Based on scanning electron microscopy (SEM) and infrared spectroscopy, it is deduced that ILs can destroy the cellulose structure in the cell wall. However, these were not sufficient to comprehensively understand the mechanism of ILs. Molecular dynamics (MD) simulations and Quantum Mechanical (QM) calculations have been used to study these interactions at the molecular level [10]. Consequently, these are powerful tools for exploring the mechanism of ILs in improving essential oil extraction efficiency.

The purpose of this paper, taking *Amomi fructus* essential oil as an example, is to comprehensively explore the mechanism of ILs in enhancing extraction efficiency based on the MD and QM. Firstly, based on the design of the experiment (DoE), multivariate analysis (MVA) models were developed to optimize multi-objective extraction process of essential oil. Furthermore, MILT-HD was shown to be an efficient and environmentally friendly extraction technique by calculating energy demands and environmental impact. Finally, according to the quantum chemical calculation and MD simulations, two mechanisms for ILs improving the extraction efficiency of essential oils were proposed. This work helps to understand the complex process of isolating essential oils and to also comprehensively understand the mechanism of ILs.

## 2. Experiment

### 2.1. Materials and Chemicals

*Amomi fructus* was purchased from the local Chinese medicinal pharmacy in Beijing, China. Four ionic liquids containing 1-butyl-3-methylimidazolium Bromide ([C4mim]Br, Lot number KYFCC03), 1-Octyl-3-methylimidazolium Bromide ([C8mim]Br, Lot number ATW598), 1-butyl-3-methylimidazolium chloride ([C4mim]Cl, Lot number KCDGP56), 1-allyl-3-methylimidazolium chloride ([Amim]Cl, Lot number L1916039) were bought from Innochem Company (Beijing, China). Other reagents used for gas chromatography-mass spectrometry (GC-MS) analysis were provided by Tianjin Kermel Chemical Reagent Co., Ltd., (Tianjin, China). The water used in the extraction process was deionized.

### 2.2. Extraction Process

For microwave assisted ionic liquids treatment followed by hydro-distillation (MILT-HD), a domestic Glanz microwave oven (Glanz electrical appliance industrial Co., Ltd., Foshan, China) was used for experiments, with maximum irradiation power of 800 W and total input power of 1300 W. In this procedure, the extraction process includes two stages: MILT step and HD process. In the first step, the *Amomi fructus* (10 g) and ILs were weighted accurately, and then introduced into a 250 mL reaction flask. The reaction flask was then symmetrically placed into the microwave oven and connected to the Clevenger apparatus through a modified hole on the top of the microwave oven. According to the DoE, the sample mixtures were preprocessed with ILs at different operating conditions. In the second stage, 60 mL deionized water was added into the reaction flask. Furthermore, a dark slurry, which was obtained by the first step, was sufficiently mixed with deionized water. Then the reaction flask was put in an electric stove (H22-X3, Hangzhou Joyoung electrical appliance Industrial Co., Ltd., Hangzhou, China), and connected to the Clevenger equipment. The electric stove was set to 100 °C for hydro-distillation process.

For the hydro-distillation (HD) procedure, *Amomi fructus* (10 g) was added into the reaction flask. Then the reaction flask connected to the Clevenger apparatus was heated in an electric stove. This process was performed at 100 °C until no more essential oil was collected. The essential oil was dehydrated with anhydrous sodium sulfate, and then stored in amber-colored vials at 4 °C until use. The yield of essential oil was calculated by Equation (1).

(1) $$Y_{eo}\%=\frac{volume\;of\;essential\;oil(mL)}{weight\;of\;sample\;(g)}\times 100\%$$

### 2.3. Experimental Design of Extraction Process

#### 2.3.1. Kinetic Model

In this study, a first-order kinetic model was used to explore the extraction process of essential oil. The relevant mathematical equations were shown as follows:

(2) $$Y_{t}=Y_{eo}[1-\exp(-k\times t)]$$

where *Y_t_* and *Y_eo_* (%, mL·g^−1^) represent the yield of essential oil at time *t* (min) and at equilibrium, respectively. *k* (% min^−1^) is the mass transfer coefficient of the entire extraction process.

#### 2.3.2. DoE for MILT-HD

According to the previous study, the stage of MILT is a critical operation unit in the extraction process. Three variables, including ILs ratio (*X*_1_), microwave irradiation time (*X*_2_), and microwave irradiation power (*X*_3_), were identified as critical process parameters (CPPs). Consequently, it is necessary to optimize these three CPPs in this stage. A Box-Behnken design was used to investigate the effects of the CPPs on the response factors. According to the preliminary experiments, the ILs ratio (*X*_1_), microwave irradiation time (*X*_2_), and microwave irradiation power (*X*_3_) were in the range 50–90%, 2–4 min, and 80–240 W, respectively. The DoE for 15 experiments is exhibited in Table 1. In previous research [8,9], only essential oil yield was a response factor, which could not systemically provide insight into the extraction process. Hence, to comprehensively investigate the effects of CPPs on the extraction process, the *Y_eo_*, the yield of essential oil at 50 min (*Y*_t50_), and *k* were response factors in this work.

Based on the DoE, a multivariate analysis (MVA) model was developed and evaluated by analysis of variance (ANOVA). According to the multivariate modeling technique, response surface methodology (RSM) was developed to explore the main effects, interaction effects, and quadratic effects of the three CPPs. A multi-objective optimization method was used to determine the optimum operating parameters for increasing the extraction efficiency of essential oil.

### 2.4. GC-MS Analysis of Essential Oil

GC-MS spectra were recorded using GCMS-QP2010 Ultra spectrometer (Shimadzu Corporation, Kyoto, Japan) consisting of a gas chromatograph equipped with a WM-5MS capillary column (30 m × 0.25 mm × 0.25 μm film thickness, Welchrom corporation, Shanghai, China) and a mass selective detector.

The essential oil samples stored in the dark were diluted with ethyl acetate (1:100, *v*/*v*) before GC-MS analysis. The GC-MS analysis parameters were set to as follows: injector temperature 250 °C, injection volume 1 μL, injection method split ratio 1:20, helium gas flow rate 1 mL·min^−1^. The column temperature was set at 60 °C for 2 min, then increased to 120 °C at a rate of 5 °C·min^−1^, then increased again to 200 °C at a rate 10 °C·min^−1^, and finally to 250 °C at a rate 20 °C·min^−1^ for 1 min. The ion source temperature and scan range were set at 220 °C and 30–500 amu, respectively. The identification of essential oil constituents was implemented by comparing literature data and comparing their mass spectra fragmentation patterns with those of similar compounds stored in library NIST08.

### 2.5. Fourier Transforms Infrared Spectroscopy (FTIR)

After being extracted by different methods, the samples were dried, milled, mixed with potassium bromide, and pressed into tablets for infrared scanning. In the pure potassium bromide background, the IR spectra of test samples can be obtained by using a spectrophotometer (Perkinelmer, PerkinElmer enterprise management (Shanghai) Co., Ltd., Shanghai, China) within 4000–400 cm^−1^. According to the IR spectra, the functional groups or chemical bonds of various samples could be qualitatively identified, which was beneficial for further exploring the alterations in the chemical structures of *Amomi fructus* samples.

### 2.6. Scanning Electron Microscopy (SEM)

The *Amomi fructus* samples extracted by different methods were dried in an oven. The *Amomi fructus* sample and dried samples were fixed on a metal disc and put into an ion sputtering apparatus (SC7620, Quorum, Laughton, East Sussex, UK). Then, the surface morphological changes of samples were observed using a scanning electron microscope (Quanta250, FEI, Hillsboro, OH, USA) with high vacuum condition at a voltage of 15.0 kV.

### 2.7. Calculation Method of Quantum Chemical

In order to explore the mechanism of action of ILs, cellobiose as a model molecule was used to investigate the cleavage of the cellulose chain. The structures of all chemical compounds in this paper were optimized by Gaussian 09 package [11] at the B3LYP/6-31G(d) level using a computer equipped with a Ryzen 2700 AMD CPU and 32G RAM. At the same level of density functional theoretical (DFT) method, intrinsic reaction coordinate (IRC) was used to search and construct the minimum-energy path. It verified the connectivity between a given transition state and the reaction/product minima [12]. The interaction energy data, atoms in molecules (AIM), and reduced density gradient (RGD) were calculated at the M062X/6-311+G(d,p) level based on the Multiwfn code [13].

### 2.8. Molecular Dynamics Simulation

**Figure 1:**
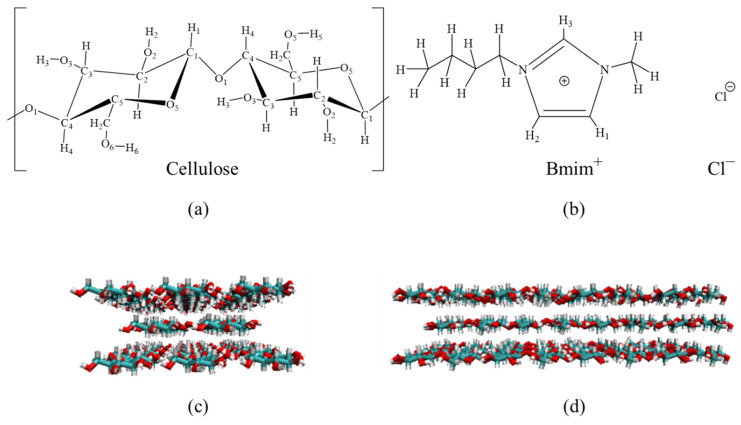
(**a**) The structures of C4mimCl; (**b**) the structures of glucose units in the simulation. (**c**) Front view of cellulose model; (**d**) Side view of the cellulose model.

A cellulose bunch composed of eight I_β_ chains, each with eight residues, was constructed by Cellulose-Builder [14], as shown in Figure 1. The Glycam06 force filed [15] parameters were applied for the cellulose bunch, while the SPC/E model [16] was used for water. For the ILs, the force field parameters for [C4mim]Cl were taken from the general AMBER force field (GAFF) [17]. For the molecular dynamic (MD) simulation, the cellulose bunch was placed in the center of a cube box filled with 1000 ionic liquids pairs. The long-range electrostatic interactions were calculated by Particle-mesh Ewald summation, and the cutoff radius was set to 1.2 nm. The cutoff value for Van der Waals forces was also 1.2 nm. Periodic boundary conditions were used to simulate the bulk system in *x*, *y*, and *z* directions. In order to remove the possible coordinate collisions, the system was minimized by the steepest descent method until the maximum force between atoms was under 500 kJ·mol^−1^·nm^−1^. Then, the system was carried out under canonical ensemble (NVT) with a temperature at 373 K. The NVT equilibrium time was 100 ps. Next, the NPT ensemble was carried out at 10 ns equilibration. The MD run of 1000 ns was performed in the NPT ensemble, and the time step was 0.002 ps. All covalent bonds in the MD system were constrained through the LINCS algorithm [18].

### 2.9. Data Analysis

The first-order kinetics models were fitted by the MATLAB 2009a platform (MathWorks, Nadick, MA, USA). Based on Design-Expert 8.0 (State-Ease, Inc., Minneapolis, MN, USA), the MVA models were developed. All experimental data were expressed as means ± standard deviations.

## 3. Results and Discussion

### 3.1. Determination of the Key Operating Parameters Affecting the Extraction Process

#### 3.1.1. Key Parameters Affecting the *Y*_t50_

According to Supplementary materials (Figure S1), the extraction efficiency was higher with [C4mim]Cl. In the previous study, the [C4mim]Cl has low toxicity [19], and it has a less harmful effect on the environment [20]. Consequently, in this work, [C4mim]Cl performed the best and was selected as the optimal IL for isolating *Amomi fructus* essential oil.

**Figure 2:**
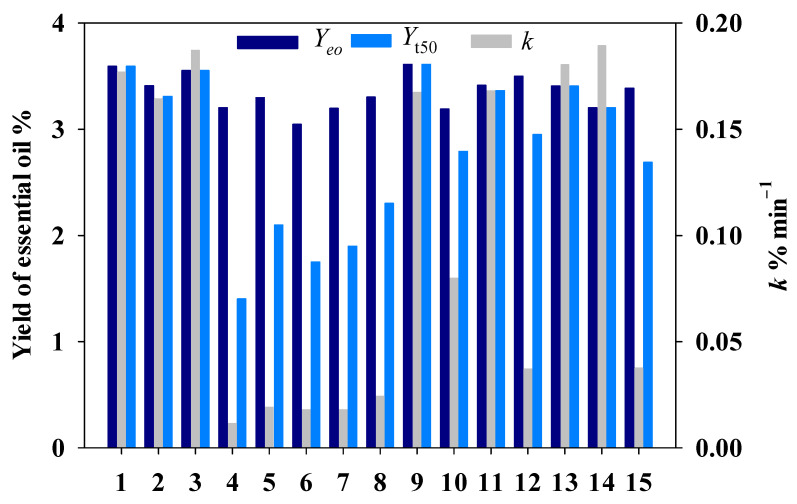
The results for 15 experiments of MILT-HD.

Based on the DoE, *Amomi fructus* was subjected to MILT process. Then the essential oils were isolated through the HD process. According to Equations (1) and (2), three response factors could be calculated as shown in Figure 2. It could be seen that *Y*_t50_ and *k* show obvious fluctuations under different experimental conditions, while the change of *Y_eo_* was not obvious and within 3.05–3.61%.

MVA models were used to explore responses based on the three key variables. The results with a summary of fit for quadratic models were listed in Table 2. It could be seen from Table 2 that the determination coefficient (R^2^) and adj-R^2^ were equal to 0.9857 and 0.9713, respectively. The *p*-value of the model was less than 0.001. It indicated that the relationship between independent variables and *Y_eo_* was extremely significant. Moreover, the *p*-value of “lack of fit” was 0.3947, indicating that the lack of fit was not significant. It implied that the MVA model was well fitted [21]. Adeq precision means that the signal-to-noise ratio and value of index larger than 4 is desirable [22]. In this paper, the Adeq precision was 25.42. In summary, the MVA model had good accuracy. The MVA model was expressed by code as follows:

(3) $$Yeo=3.44+0.18X_{1}+0.58X_{2}+0.58X_{3}-0.16X_{2}\times X_{3}-0.43X_{1}{}^{2}-0.55X_{2}{}^{2}-0.23X_{3}{}^{2}$$

In this paper, the model terms with a *p*-value less than 0.05 were considered significant terms. From Table 2, it could be found that linear terms of *X*_1_, *X*_2_, and *X*_3_, the interactions term of *X*_2_*X*_3_, and the quadratic terms of *X*_1_^2^, *X*_2_^2^, and *X*_3_^2^ reached statistical significance. It was found that all terms of interactions between various operating parameters had negative impacts on the *Y*_t50_. The terms with a large regression coefficient and small *p*-value indicated more significant response factors [23]. According to the MVA model, the linear terms of *X*_2_ and *X*_3_ had the largest effect on the *Y*_t50_. It revealed that a big variation of *Y*_t50_ could be achieved by changes in microwave irradiation time (*X*_2_) and microwave irradiation power (*X*_3_).

#### 3.1.2. Analysis of Response Surface for the *Y*_t50_

**Figure 3:**
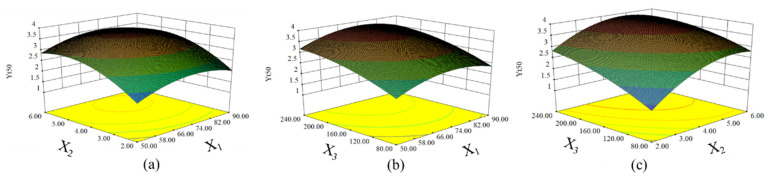
Response surface graphs of *Y*_t50_. (**a**) Interaction of ILs ratio and microwave irradiation time; (**b**) Interaction of ILs ratio and microwave power; (**c**) Interaction of microwave irradiation time and microwave power.

Surface response plots, as a three-dimensional plot, were utilized to visualize the MVA model. As exhibited in Figure 3a, the *Y*_t50_ would increase and then decrease with increasing [C4mim]Cl mass concentration at a given irradiation power. It is likely to improve the dissolving capacity of the cellulose by increasing the mass concentration of ILs. It may be conducive to enhance the permeability of cell walls, resulting in a higher release of essential oils [8]. However, with a greater increase of IL concentration, the viscosity of ILs would increase, which would reduce the mass transfer of the extraction process. Consequently, the extraction efficiency may decrease [24]. With the increase of power, *Y*_t50_ presented an upward trend. Since IL has excellent microwave absorbing capacity, it helped increase the ability of ILs to penetrate plant tissue with increasing microwave power within a certain range, which resulted in destruction of the cell wall and enhancement of mass transfer [25]. As shown in Figure 3c, the *Y*_t50_ rapidly increased with increasing microwave irradiation time. However, further prolonging microwave irradiation time slightly reduced *Y*_t50_. There is no doubt that a longer microwave irradiation time was beneficial for dissolving the cell wall [9]. However, the raw material temperature could dramatically rise with prolonged microwave irradiation time, which led to some liable volatile components being lost or decomposition of some thermosensitive constituents [25]. Consequently, the appropriate optimal operating parameters should be within a reasonable range.

#### 3.1.3. Multiple Response Optimization and Verification

In this work, not only was the *Y*_t50_ optimized, but also the extraction efficiency *k* and *Y_eo_* were optimized. For the *k* and *Y_eo_*, the results for quadratic models were listed in Tables S1 and S2. In order to optimize three responses simultaneously, the desirability function [23] in the Design-Expert software was used to obtain optimal process conditions. According to the models, the optimal conditions could be easy to acquire, as shown in Table 3. Under the optimum process parameters, three experimental data were applied to verify the suitability of the reduced quadratic models. It can be seen from Table 3 that the relative errors between predicted and experimental results were small. It suggested that the reduced quadratic models were reasonable and reliable.

### 3.2. Comparison of Extraction Efficiency

**Figure 4:**
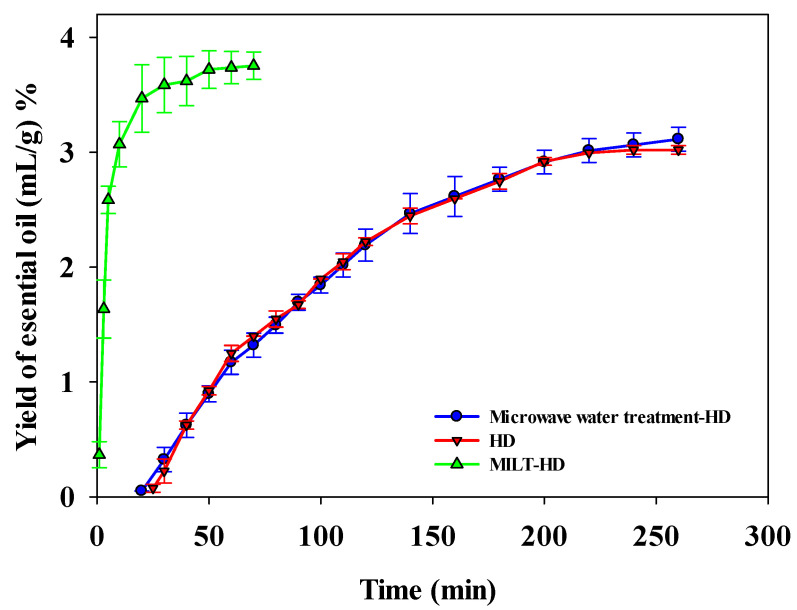
The extraction efficiency of essential oil obtained by different methods.

In order to compare the extraction efficiency of the two extraction methods, the MILT-HD method was used to extract essential oil according to the above optimal process parameters. The result could be seen Figure 4. It could be found that the MILT-HD could quickly reach the extraction equilibrium point. The yield of essential oil extracted by MILT-HD was 3.753% ± 0.119. However, the yield of essential oil extracted by HD was 3.020% ± 0.037, and this method demanded a longer extraction time. It was shown that MILT-HD was an efficient extraction technique. To eliminate the impact of the microwave process, the microwave water treatment -HD (MWT-HD) process was implemented using similar optimal parameters from the MILT-HD optimization process. It can be seen from Figure 4 that the extraction efficiency of MWT-HD was similar to that of HD. Consequently, the transient microwave radiation in this work had no significant effect on the extraction process of essential oils.

Since energy demands represent the major expenditure in most chemical process [26], the energy consumptions and CO_2_ emissions in different extraction methods were calculated. Table 4 summarized the energy demands and CO_2_ emissions during the essential oil extraction using MILT-HD and HD methods. It could be observed that MILT-HD consumed around one-third of the electrical energy of HD, but MILT-HD approach could isolate more essential oil. Based on the reference, for 1 kW·h electricity consumed, 800 g CO_2_ would be released into the atmosphere [27]. In this paper, the HD method produced 1920 g CO_2_ during the extraction process of essential oils. In the MILT-HD process, the yield of essential oil per kilowatt hour was 0.0514 mL/g/(kW·h). However, this value in HD process was only 0.0126 mL/g/(kW·h). It was shown that the MILT-HD had higher extraction efficiency compared to the HD process. The above analysis demonstrated that the MILT-HD is an efficient and environmentally friendly extraction technique.

### 3.3. GC-MS Analysis

GC-MS were applied to compare the component difference of essential oil between the conventional HD and MILT-HD. The identified chemical compositions were listed in Table 5. It could be seen from Table 4 that principal components were bornyl acetate, camphor, camphene, limonene, myrcene, borneol, and pinene. This result was consistent with the previous literature [28,29]. It was found that 12 components in HD accounted for 97.35% of the total essential oil, and in the MILT-HD, the 12 components accounted for 94.42%. At the same time, it was found from Table 5 that the relative contents of the main components extracted by MILT-HD did not change compared to the essential oil extracted by the HD process. According to the GC-MS analysis, the result demonstrated that ILs assisted extraction had little effect on the components of essential oil. This conclusion was in line with other results in the published literature [30].

### 3.4. Mechanism Analysis of ILs

#### 3.4.1. Cleavage Cellulose Chain

**Figure 5:**
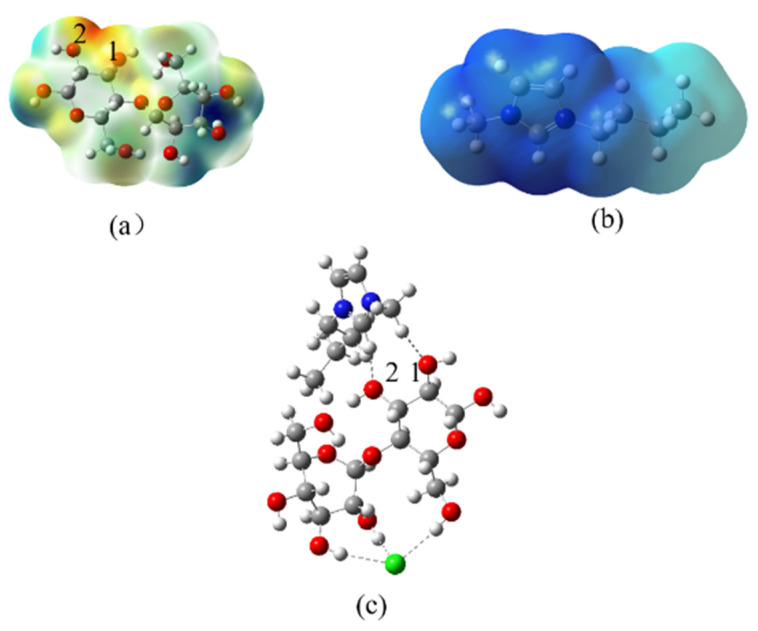
(**a**) Geometrics and electrostatic potential (ESP) surfaces of cellobiose (Carbon: gray; oxygen: red; hydrogen: white), (**b**) Geometrics and EPS of [C4mim]^+^ (Carbon: gray; hydrogen: white; nitrogen: blue), (**c**) geometrics of interaction with [C4mim]Cl-cellobiose (Carbon: gray; oxygen: red; hydrogen: white; nitrogen: blue; bromine: green).

In order to further explore the mechanism of improving the extraction efficiency of ILs, the DFT calculation was implemented to obtain some important information on cellulose–ILs interactions. Given that cellulose contains several hundred to many thousands of *β* (1–4) linked *D*-glucose units [31], cellobiose was selected as a model molecule for investigating the cleaver cellulose chain. Figure 5 shown the optimized geometries and electrostatic potential surfaces of [C4mim]Cl and cellobiose. In this figure, the red color meant a negative potential surface, while the blue color represented the positive ones. It could be observed that electrostatic potentials of all the regions around [C4mim]^+^ are positive, and the positive charges were mainly located on the heterocyclic rings. In the cellobiose structure, the negative charges were mainly located on the oxygen atoms, while the hydrogen atoms of the hydroxyl group were positive charges. In Figure 5a, the maximum negative charge position was located on the area between 1 and 2 oxygen atoms, indicating that the cation of ILs would interact with cellobiose in this area. This conjecture was verified by DFT calculation for ILs interacting with cellobiose, as shown in the Figure 5c. In Figure 5c, the anions tend to form H-bonds with cellobiose, and the H receptor is relatively close. Based on the above analysis, the reaction bonding sites between ILs and cellobiose were determined.

**Figure 6:**
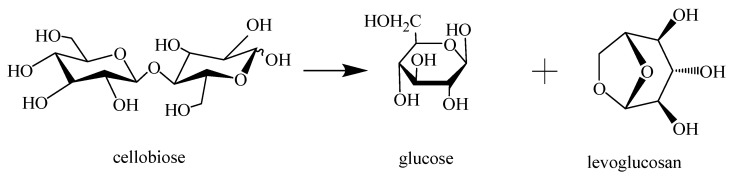
The reaction of the cleavage of cellobiose in [C4mim]Cl.

**Figure 7:**
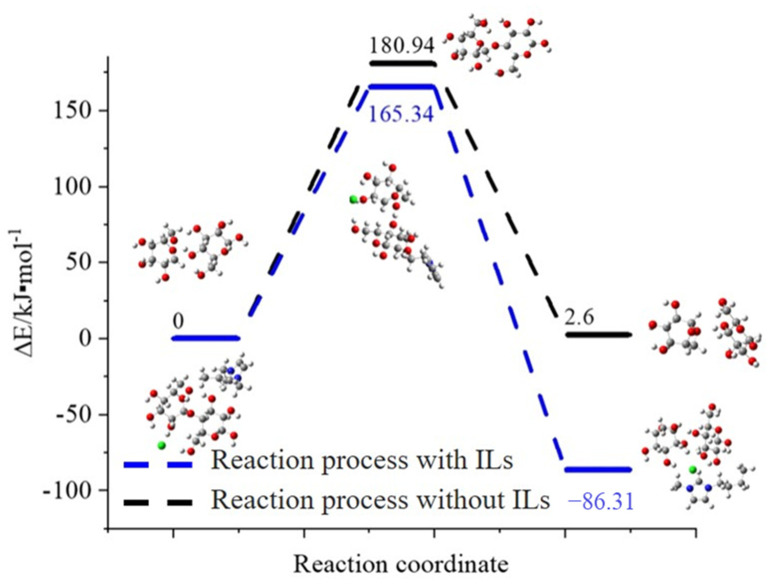
Structure diagrams and relative energy for cleavage of cellobiose catalyzed by ILs and without ILs. (Carbon: gray; oxygen: red; hydrogen: white; nitrogen: blue; bromine: green).

Based on the evidence from DFT calculations [12], the routes for cleavage of cellobiose were catalyzed by [C4mim]Cl, as shown in Figure 6. The geometries of reactant, TS, and product were optimized. The corresponding energy diagrams were exhibited in Figure 7. When the reaction was catalyzed by [C4mim]Cl, the products, including glucose and levoglucosan, were formed through the reaction proceeding through a transition state. In the TS, a C–O bond of cellobiose would break first, and then a C–O bond of levoglucosan would form. In this reaction process, about 165.34 kJ·mol^−1^ energy was needed to cross the energy barrier, and 86.31 kJ·mol^−1^ was exothermic. During the whole reaction process, ILs did not break and generate chemical bonds, indicating that ILs played a role as a catalyst to promote the cleavage reaction. However, in the reaction process without ILs, a slightly higher energy barrier must be crossed, and the whole reaction step was endothermic with 2.6 kJ·mol^−1^. Since ILs can reduce the reaction energy barrier to a certain extent and release heat, the reaction process with ILs occurred relatively easily. It indicated that ILs were more likely to damage cellulose chains in plant cell walls than in the absence of ILs. One of the mechanisms for ILs improving the extraction efficiency of essential oil is that ILs reduced the cellulose chains, which resulted in changing the cellulose structure. It is good for increasing the permeability of the cell wall and facilitating the release of essential oil.

#### 3.4.2. Structure Change of Cellulose in the BmimCl and Water

**Figure 8:**
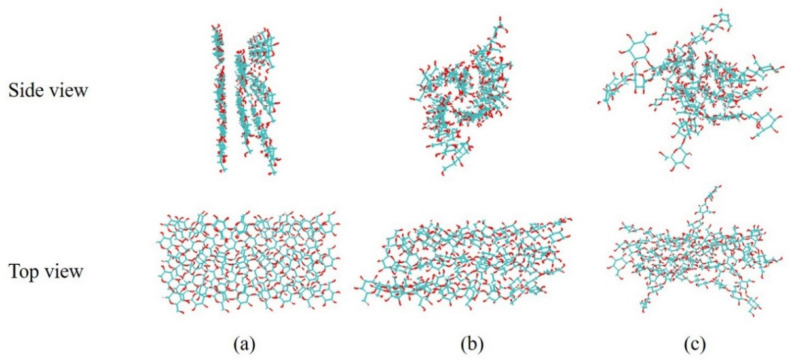
Configuration of the cellulose model before and after 1000 ns simulation. (**a**) 0 ns; (**b**) 1000 ns in the water; (**c**) 1000 ns in the ILs.

**Figure 9:**
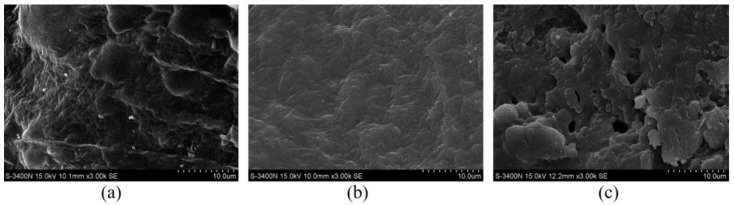
SEM images of tested samples. (**a**) Sample of raw material; (**b**) Sample of HD; (**c**) Sample of MILT-HD.

After the 1000 ns molecular simulations, the initial conformation and ending conformations of the cellulose model in [C4mim]Cl and water were shown in Figure 8. It can be seen that the cellulose has been obviously twisted in the water, which is caused by the hydrogen bonds within and between the chains in the fibrils [32]. However, the structure of cellulose was relatively complete, which was consistent with the results of SEM, as shown in Figure 9. In [C4mim]Cl, the structure of cellulose has been destroyed, which shows evident dissolving behavior. It can be seen from Figure 9 that the morphological structure of MILT-HD sample was significantly different from the raw material. Most of the cell walls were disrupted or broken. It is conducive to essential oils rapidly leaking from cells without a slow permeation [33], and to promote the complete escape of the essential oil from cells [34].

**Figure 10:**
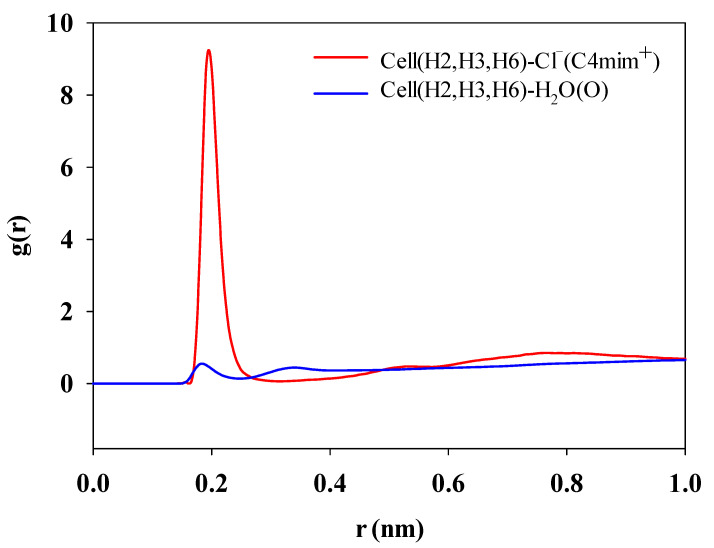
Radical distribution function of the negatively charged atoms of different anions around the cellulose hydrogens in the hydroxyl group.

**Figure 11:**
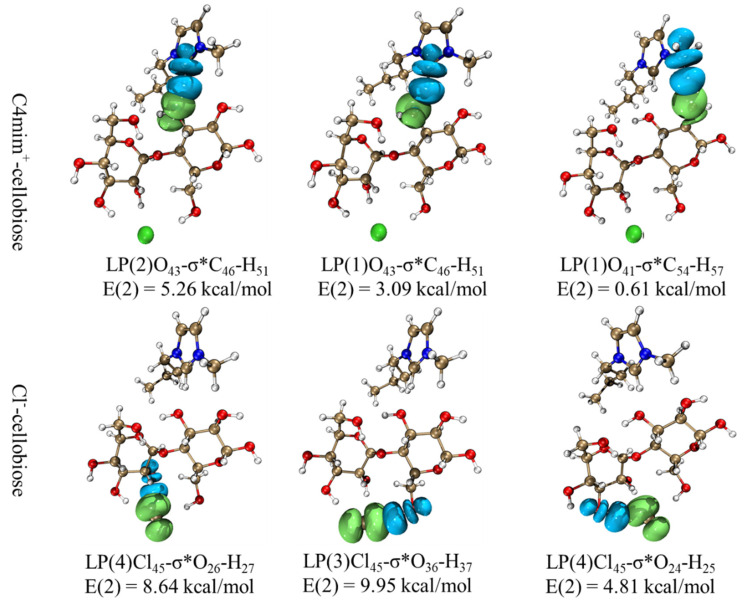
3D overlap images for donor-acceptor orbital interactions. (Carbon: gray; oxygen: red; nitrogen: blue; bromine: green; hydrogen: white).

To quantitatively characterize the interaction, the interaction energies of cellulose with [C4mim]Cl and water were calculated. The interaction energy is the sum of the Coulomb force (*E*_coul_) and the Lennard-Jones potential (*E*_L-J_). *E*_coul_ and *E*_L-J_ refer to electrostatic interaction and van der Waals interaction between solvent and cellulose, respectively. The results are listed in Table 6. It can be found that the interaction energy between [C4mim]Cl and cellulose chains was equal to −6027.43 kJ/mol. However, the total interaction energy between water and cellulose chains was equal to −10,477.92 kJ/mol. It indicated that the interaction between [C4mim]Cl and cellulose was much smaller than that of [C4mim]Cl. It can be seen from Figure 10 that water was not concentrated in its distribution around the cellulose. Consequently, it is difficult to destroy the cellulose sheets interaction, which includes many hydrogen bonds and strong van der Waals interaction. In ionic liquids, the large interaction energies between [C4mim]Cl and cellulose was beneficial to undermine the cellulose intersheet interactions. According to the NBO analysis, it can be observed that there were track overlaps between [C4mim]Cl and cellulose, as shown in Figure 11. The corresponding second-order perturbation E(2) values of anion were generally greater than that of cations, which was also in line with Table 6. The large E(2) means a strong interaction between the anion with cellulose. As seen from Table 6, the electrostatic interaction energy between anions and cellulose was large. However, the interaction between cations and cellulose mainly behaved as van der Waals force. Under the combined action of van der Waals and electrostatic forces, cation and anion in ILs work synergistically to destroy cellulose [31].

#### 3.4.3. H-Bond Change in the Cellulose Dissolving Process

**Figure 12:**
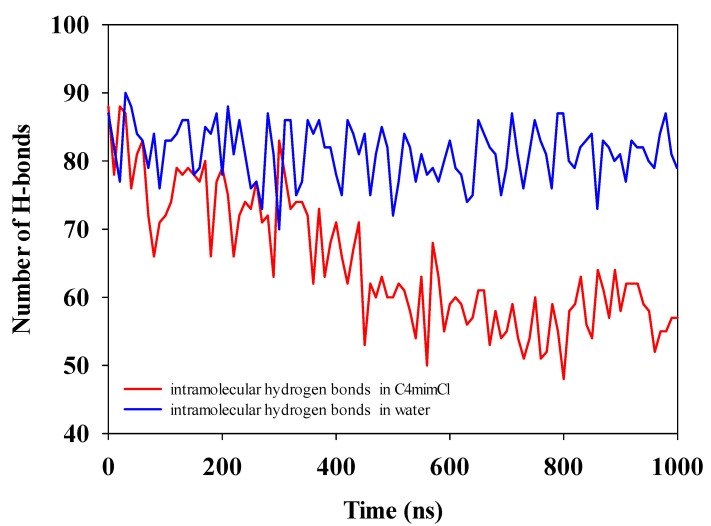
The intramolecular hydrogen bonds in water and [C4mim]Cl.

The hydrogen bond network is one of the most important factors for the natural resistance to deconstruction of cellulose in plant cell walls. In this paper, the H-bond change was used as an indicator to evaluate the dissolution of cellulose in solution. The criteria for hydrogen bond formation need to meet two criteria used by Gromacs: the donor-acceptor distance should be less than 0.35 nm, and hydrogen-donor-acceptor angle should be smaller than 30° [32]. The change traces of H-bond network in cellulose were shown in Figure 12. There is almost no obvious change in the intramolecular hydrogen bonds of cellulose in water, while in the [C4mim]Cl, an evident decreasing tendency was observed. It indicated that the crystal structure of cellulose has been destroyed and gradually dispersed in [C4mim]Cl. Based on the published paper [35], the ILs easily formed hydrogen bonds with hydroxyl groups in cellulose, thus destroying the network hydrogen bond structure in cellulose.

**Figure 13:**
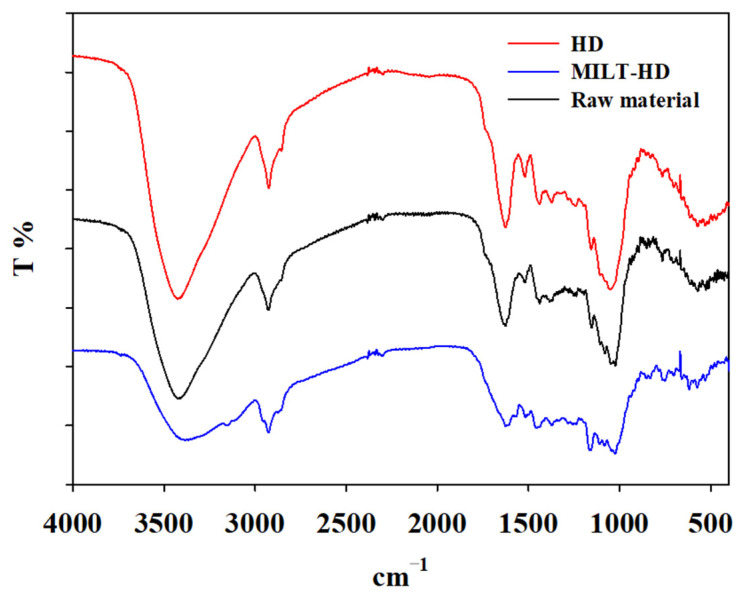
FTIR spectra of tested samples.

According to the IR spectra, it can be seen from Figure 13 that the signal intensity of the absorbance bands was not obviously changed in the HD sample as against those of raw material *Amomi fructus*. It demonstrated that chemical structures of carbohydrate compounds were unbroken, indicating the cell wall kept integrity after HD process. But, in the IR spectra of sample by MILT process, the absorbance intensity at 3424 cm^−1^ (OH stretch) decreased apparently, which was in good agreement with the results of molecular dynamics simulation. It revealed that IL could destroy the network of hydrogen bonds between the carbohydrate hydroxyl protons [8], suggesting that IL can dissolve cellulose in the cell wall.

**Figure 14:**
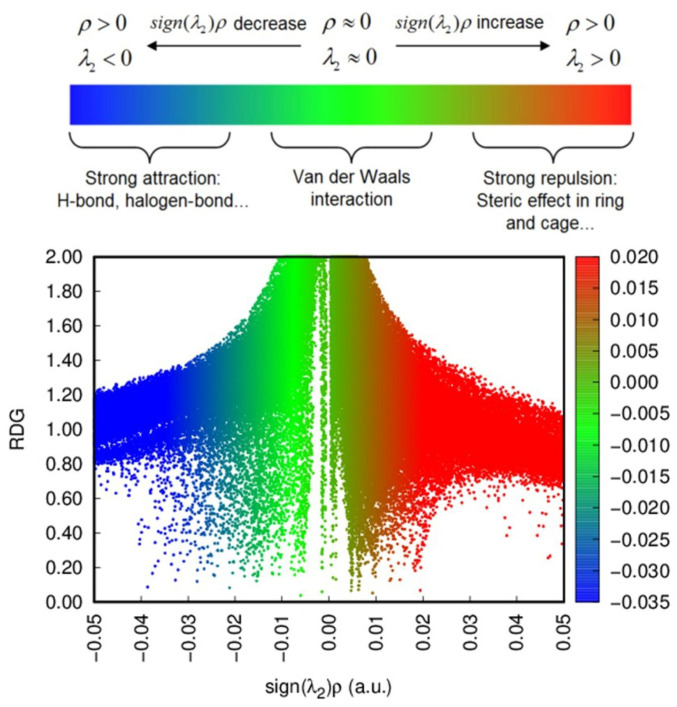
The RDG scatter plot.

In addition to forming hydrogen bonds with cellulose, ILs can also produce other non-covalent bonds. Reduced density gradient (RDG) is a feasible approach to describe the non-covalent interactions based on the electron density and its derivatives [36]. In color mapped RDG isosurfaces and scatter diagrams, as shown in Figure 14, a large negative (positive) value of sign (λ2) ρ indicates hydrogen bond (steric repulsion), which was shown in blue (red), and values close to zero represent van der Waals forces, colored in green [37]. Consequently, it was revealed that [C4mim]Cl could have a variety of strong non-covalent bonds with cellulose, which was helpful for promoting the destruction and solvation of the cellulose structure.

#### 3.4.4. Supposed Mechanism of Ionic Liquids in Improving the Extraction Efficiency

**Figure 15:**
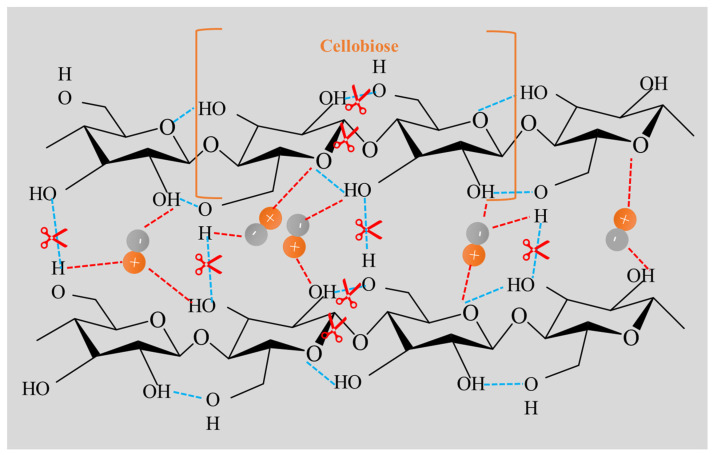
Graphic mechanism of ILs for enhancing extraction efficiency.

Based on the above analysis, the mechanism of ILs to improve the extraction efficiency of essential oils includes two aspects: ILs can facilitate the breakage of cellulose chains and reduce the length, and ILs can form non-covalent bonds with cellulose, especially hydrogen bonds, which can significantly destroy network hydrogen bond structure between cellulose sheets, as shown in Figure 15. Through the IL process, the cell wall in the plants can be breakdown. The essential oil in the plant can easily penetrate the cell wall and be released without too many barriers. Consequently, the MILT-HD approach can significantly improve the extraction efficiency of essential oil from *Amomi fructus*.

## 4. Conclusions

In this study, [C4mim]Cl was shown to be an efficient ionic liquid used in the MILT-HD process. DoEs and MVA were used to comprehensively evaluate the process of extracting essential oils. Furthermore, the operating parameters determined by multi-objective optimization accelerated the isolation of essential oil from *Amomi fructus* without obvious alteration in the essential oil components. Under the optimal operating conditions, MILT-HD approach not only improved the yield and extraction efficiency of *Amomi fructus* essential oil, but also required low energy consumption and reduces CO_2_ emissions. Based on the DFT and molecular dynamics simulations, the mechanisms for ILs improving the extraction efficiency of essential oil were comprehensively explored. One mechanism is through promotion of the cleavage of the cellulose chain. The other is destroying the network hydrogen bond structure of cellulose by non-covalent interactions.

However, there are still some limitations in this study. In this study, only the cellulose model was used for molecular dynamics and DFT studies, and other chemical components in the cell wall were ignored. In the future, a cell wall model containing lignin and other chemical components can be constructed for further research.

## Figures and Tables

**Table 1:** Box–Behnken experimental design matrix for the extraction of essential oil during the MILT process.

Runs	Process Parameters		
	*X*_1_ (%)	*X*_2_ (min)	*X*_3_ (W)
1	90	4	240
2	50	4	240
3	70	4	160
4	70	2	80
5	90	2	160
6	50	2	160
7	50	4	80
8	90	4	80
9	70	6	240
10	50	6	160
11	70	4	160
12	70	6	80
13	70	4	160
14	90	6	160
15	70	2	240

**Table 2:** ANOVA results for response surface quadratic method for *Y*_t50_.

Source	Sum of Square	DF	Mean Square	*F* Value	*p*-Value	Significance
Model	7.50	7	1.07	68.78	<0.0001	Significant
*X*_1_	0.26	1	0.26	16.82	0.0046	Significant
*X_2_*	2.67	1	2.67	171.10	<0.0001	Significant
*X*_3_	2.70	1	2.70	173.49	<0.0001	Significant
*X_2_ X*_3_	0.098	1	0.098	6.31	0.0403	Significant
*X*_1_^2^	0.70	1	0.70	44.63	0.0003	Significant
*X*_2_^2^	1.10	1	1.10	70.88	<0.0001	Significant
*X*_3_^2^	0.20	1	0.20	12.69	0.0092	Significant
Residual	0.11	7	0.016			Significant
Lack of fit	0.089	5	0.018	1.8	0.3947	Not significant
R^2^	0.9857					

**Table 3:** Predicted and experimental values of the responses obtained under the optimal extraction conditions.

	*X*_1_ (%)	*X*_2_ (min)	*X*_3_ (W)	*Y_eo_* (%)	*Y*_t50_ (%)	*k* (% min^−1^)	Desirability
Predicted	74.00	4.24	233.12	3.611	3.842	0.194	1.00
Experimental	74.00	4.25	240.00	3.753 ± 0.119	3.720 ± 0.164	0.188 ± 0.0045	
RE (%)				3.78	−3.28	−3.19	

Note: RE (%) represents relative error.

**Table 4:** Comparison of the economic value and environmental impact of different extraction approaches.

	MILT-HD		HD
	Pretreatment	Hydrodistillation	Hydrodistillation
Heating Method	Microwave	Electric stove	Electric stove
Effective electric power (W)	390	600	600
Time consumption (h)	0.0707	1.17	4
Electricity consumption (kW·h)	0.0276	0.702	2.4
Total electricity consumption (kW·h)	0.730		2.4
Yield of essential oil (mL/g)	0.0375		0.0302
Yield of essential oil per kilowatt hour (mL/g/(kW·h))	0.0514		0.0126
Environmental impact (g CO_2_ emission)	584.0		1920

**Table 5:** Key data for the major components of the *Amomi fructus* essential oil.

No.	Components	Molecular Formula	Relative Contents (%)	
			HD	MILT-HD
1	Pinene	C_10_H_16_	1.530	1.620
2	Camphene	C_10_H_16_	8.333	7.323
3	Myrcene	C_10_H_16_	2.927	2.853
4	Phellandrene	C_10_H_16_	0.197	0.200
5	Limonene	C_10_H_16_	7.540	7.437
6	Terpinolene	C_10_H_16_	0.110	0.280
7	Linalool	C_10_H_18_O	0.467	0.403
8	Camphor	C_10_H_16_O	29.183	29.477
9	Borneol	C_10_H_18_O	2.517	2.347
10	Bornyl acetate	C_12_H_20_O_2_	44.390	41.343
11	Caryophyllene	C_15_H_24_	0.087	0.597
12	Cadinene	C_15_H_24_	0.070	0.543
Total%			97.35	94.42

**Table 6:** Interaction energies of cellulose with [C4mim]Cl and water.

*E* (kJ/mol)	Water	C4mim^+^	Cl^−^
*E*_coul_	−5346.40	−1058.52	−6698.34
*E*_L-J_	−681.03	−3302.19	581.132
*E*_total_	−6027.43	−4360.71	−6117.21

## Data Availability

The data presented in this study are available on request from the corresponding author. The data are not publicly available due to privacy.

## Supplementary Materials

The following supporting information can be downloaded at: https://www.mdpi.com/article/10.3390/molecules27175515/s1, Figure S1: Effects of various ILs on the essential oil yield (extraction process was implemented as follows: irradiation power 20%, irradiation time 4 min, mass the concentration of ILs 70% for the MILT process. Table S1: ANOVA results for response surface quadratic method for *k*. Table S2: ANOVA results for response surface quadratic method for *Y_eo_*.

## Sample Availability

Samples of the compounds are not available from the authors.
