# Supplementary material for: Exploring the Mechanism of Ionic Liquids to Improve the Extraction Efficiency of Essential Oils Based on Density Functional Theory and Molecular Dynamics Simulation
Source: Molecules. 2022 Aug 27;27(17):5515. doi: 10.3390/molecules27175515 (PMC9457939; doi:10.3390/molecules27175515)
Supplement: Supplementary file 1 [file molecules-27-05515-s001.zip › molecules-1892128-supplementary.pdf]

Supplementary materials

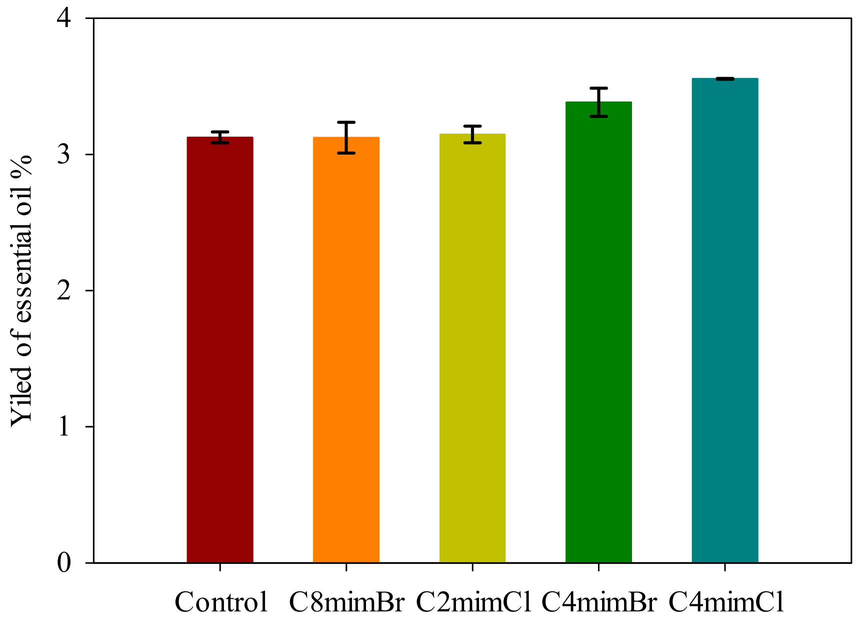

Figure S1. Effects of various ILs on the essential oil yield (extraction process was implemented as follows: irradiation power 20%, irradiation time 4 min, mass the concentration of ILs 70% for the MILT process

Table S1. ANOVA results for response surface quadratic method for *k*

Source	Sum of square	DF	Mean square	*F* value	*p*-value	Significance
Model	0.071	5	0.014	10.37	0.0016	Significant
X_2	0.019	1	0.019	13.77	0.0048	Significant
X_3	0.026	1	0.026	19.00	0.0018	Significant
X_1^2	0.0044	1	0.0044	3.26	0.1045	Not Significant
X_2^2	0.017	1	0.017	12.21	0.0068	Significant
X_3^2	0.0085	1	0.0085	6.19	0.0345	Significant
Residual	0.012	9	1.365E-003			Significant
Lack of fit	0.012	7	1.727E-003	17.83	0.0046	Not significant
R^2	0.8521					

Table S2. ANOVA results for response surface quadratic method for Y_{eo}

Source	Sum of square	DF	Mean square	*F* value	*p*-value	Significance
Model	0.30	5	0.061	6.30	0.0088	Significant
X_1	0.038	1	0.038	3.93	0.0786	Not significant
X_2	0.041	1	0.041	4.22	0.0702	Not significant
X_3	0.079	1	0.079	8.21	0.0186	Significant
X_1^2	0.10	1	0.10	10.79	0.0095	Significant
X_2^2	0.052	1	0.052	5.37	0.0456	Significant
Residual	0.087	9	0.0097			Significant
Lack of fit	0.073	7	0.010	1.53	0.4514	Not significant
R^2	0.7779					
